# The Relationship between Sarcopenia and Respiratory Muscle Weakness in Community-Dwelling Older Adults

**DOI:** 10.3390/ijerph182413257

**Published:** 2021-12-16

**Authors:** Tomoyuki Morisawa, Yota Kunieda, Shingo Koyama, Mizue Suzuki, Yuma Takahashi, Tomokazu Takakura, Yuta Kikuchi, Tadamitsu Matsuda, Yuji Fujino, Ryuichi Sawa, Akihiro Sakuyama, Masakazu Saitoh, Tetsuya Takahashi, Toshiyuki Fujiwara

**Affiliations:** 1 Department of Physical Therapy, Juntendo University, 3-2-12 Hongo, Bunkyo-ku, Ochanomizu Center Building 5F, Tokyo 113-0033, Japan; t.matsuda.ye@juntendo.ac.jp (T.M.); y.fujino.pb@juntendo.ac.jp (Y.F.); r.sawa.ia@juntendo.ac.jp (R.S.); a.sakuyama.ep@juntendo.ac.jp (A.S.); m.saito.tl@juntendo.ac.jp (M.S.); te-takahashi@juntendo.ac.jp (T.T.); t-fujiwara@juntendo.ac.jp (T.F.); 2 Department of Rehabilitation Medicine, Juntendo Tokyo Koto Geriatric Medical Center, 3-3-20 Shinsuna, Koto-ku, Tokyo 136-0075, Japan; y.kunieda.wz@juntendo.ac.jp (Y.K.); s.koyama.dh@juntendo.ac.jp (S.K.); m.suzuki.mh@juntendo.ac.jp (M.S.); y.takahashi.ng@juntendo.ac.jp (Y.T.); t-takakura@juntendo.ac.jp (T.T.); 3 Department of Rehabilitation Medicine, Juntendo University Hospital, 2-1-1 Hongo, Bunkyo-ku, Tokyo 113-0033, Japan; ytkiku@juntendo.ac.jp; 4 Department of Rehabilitation Medicine, Juntendo University Graduate School of Medicine, 2-1-1 Hongo, Bunkyo-ku, Tokyo 113-8421, Japan

**Keywords:** sarcopenia, respiratory muscle weakness, older adults, physical performance, physical activity

## Abstract

An association between respiratory muscle weakness and sarcopenia may provide a clue to the mechanism of sarcopenia development. We aimed to clarify this relationship among community-dwelling older adults. In total, 117 community-dwelling older adults were assessed and classified into 4 groups: robust, respiratory muscle weakness, sarcopenia, and respiratory sarcopenia. The respiratory sarcopenia group (12%) had a significantly higher percentage of males and had lower BMI, skeletal muscle index, skeletal muscle mass, phase angle, and oral function than the robust group (32.5%). All physical functions were significantly lower. The respiratory muscle weakness group (54.7%) had a significantly lower BMI and slower walking speed, compared with the robust group. The sarcopenia group (0.8%) was excluded from the analysis. The percent maximum inspiratory pressure was significantly lower in both the respiratory muscle weakness and respiratory sarcopenia groups, compared with the robust group. Almost all participants with sarcopenia showed respiratory muscle weakness. In addition, approximately 50% had respiratory muscle weakness, even in the absence of systemic sarcopenia, suggesting that respiratory muscle weakness may be the precursor of sarcopenia. The values indicating physical function and skeletal muscle mass in the respiratory muscle weakness group were between those in the robust and the respiratory sarcopenia groups.

## 1. Introduction

Interest in sarcopenia, the age-related loss of skeletal muscle mass and function, is growing considerably. A recent systematic review and meta-analysis reported that the prevalence of sarcopenia in community-dwelling older adults is 10% [1,2,3]. Sarcopenia is a disorder characterized by progressive and generalized loss of skeletal muscle mass and function, and it is associated with increased adverse outcomes such as physical disability, poor quality of life, and death [4,5,6,7,8,9]. Therefore, the prevention of sarcopenia is an important task for all hospital staff involved in the care of older adults.

Typical diagnostic criteria for sarcopenia include the European Working Group on Sarcopenia in Older People [10] and the Asian Working Group for Sarcopenia (AWGS) [11] criteria. Although the cutoff values for each criterion differ, grip strength, physical function (gait speed, short physical performance battery (SPPB), and chair rise speed), and skeletal muscle mass measured by bioimpedance analysis or dual-energy X-ray absorptiometry are used to assess sarcopenia. According to the AWGS 2019, the diagnosis of sarcopenia is based on physical function (6 m walk or five-time chair stand (5SCT) test or SPPB), muscle strength (handgrip strength), and skeletal muscle mass. As sarcopenia is a systemic disease, it is likely that age-related loss of muscle mass plus low muscle strength also causes weakness in the respiratory muscles such as the diaphragm and intercostal muscles. Respiratory muscles, as well as skeletal muscles, show functional decline with age [12], and this age-related decline in respiratory muscles is called “respiratory sarcopenia” [13,14]. In recent years, maximum inspiratory pressure (MIP) has been reported to be more strongly related to skeletal muscle mass and strength than maximum expiratory muscle [15], and since MIP is also used as an indicator of ventilatory failure, it is a very important indicator of respiratory muscle strength.

Respiratory sarcopenia is a relatively new concept in this field; therefore, a consensus is yet to be reached on the definition. MIP, one of the important indicators of respiratory muscle strength, has been used to operationally define respiratory sarcopenia [13]. However, considering the mechanism of the “respiratory metabolic reflex” (muscle mass loss and muscle weakness of the respiratory muscles occur first, and exercise tolerance decreases due to decreased oxygen-carrying capacity and skeletal muscle fatigue, causing muscle weakness in the limb skeletal muscles) [16], it is clear that the loss of muscle mass plus low muscle strength causes weakness in the respiratory muscles. It is impossible to deny the possibility that the decrease in muscle mass and strength of limb skeletal muscles that occurs in sarcopenia is preceded by a decrease in muscle mass and strength of respiratory muscles. If respiratory muscle weakness is associated with sarcopenia, it may provide a clue to the mechanism of sarcopenia development.

The purpose of this study was to clarify the relationship between sarcopenia and respiratory muscle weakness among community-dwelling older adults and to conduct an exploratory study to reduce respiratory muscle weakness.

## 2. Materials and Methods

This study included 137 people aged ≥65 years who participated in a cognitive and physical health survey of older adults living in the community held in Tokyo between March 2021 and June 2021. The inclusion criteria were as follows: those who were able to walk unaided, and those who had received sufficient explanations for participation in this study and had given written consent of their own free will with full understanding. Exclusion criteria were as follows: those with a history of respiratory diseases or undergoing treatment for respiratory diseases, those with serious orthopedic diseases that would interfere with measurements, those with metal implants in the body such as cardiac pacemakers or artificial joints, and those with a diagnosis of dementia. Finally, 117 participants were included in the analysis, after excluding 10 with a history of respiratory diseases, 5 whose body composition could not be assessed due to pacemakers or artificial joints, and 5 with missing data. This study was approved by the Ethics Committee of Juntendo Tokyo Koto Senior Medical Center Hospital (approval no. G20-0016); the study was explained to the participants orally, and written consent was obtained before the study was conducted.

### 2.1. Basic Information

Age, sex, medical history (cancer, heart disease, stroke, diabetes, osteoporosis, and osteoarthritis), educational history (years of education since entering elementary school), and life function were entered into a self-administered form [17]. The Japanese version of the Montreal Cognitive Assessment was used to evaluate cognitive function [18].

### 2.2. Kihon Checklist

Life function was assessed using the Kihon checklist (KCL), which was developed by the Japan Ministry of Health, Labor, and Welfare. KCL comprised 25 yes/no questions divided into 7 domains (instrumental activities of daily living (IADL), mobility, nutrition, oral function, socialization, cognitive, and depression); a higher score was associated with increased mortality and a higher risk of needing long-term care insurance services [19,20,21].

### 2.3. Respiratory Muscle Strength

MIP, the measurement of respiratory muscle strength, was measured using respiratory muscle strength testers (IOP-01, Obata Keiki Co., Osaka, Japan). Participants were asked to make maximal inspiratory effort twice in a sitting position, and a higher value was recorded as actual MIP. The predicted MIP adjusted for age and gender were used for the predicted MIP (male: 131−0.76 × age and female: 102−0.69 × age) [22], and % MIP was the value of the actual MIP divided by the predicted MIP.

### 2.4. Oral and Cognitive Functions

Oral and articulatory functions were measured using an oral function test device (TKK 3351 digital counter, Takei Kiki Kogyo Co., Niigata, Japan). The participants were instructed to repeat each syllable (“pa,” “ta,” and “ka”) as quickly as possible for 5 s, and the number of occurrences was counted with a digital counter. We calculated each syllable separately as the number of articulations per second.

### 2.5. Skeletal Muscle Strength, Physical Function, Body Measurement, and Body Composition Assessment

Skeletal muscle strength, grip strength, and knee extensor strength were measured. Grip strength was measured twice bilaterally in a standing position with the elbow extended using a Smedley digital grip strength meter (Grip-D, Takei Kiki Kogyo Co., Niigata, Japan), and the maximum value was recorded. Knee extension muscle strength was measured twice with a hand-held dynamometer (mTasF-1, ANIMA, Tokyo, Japan) when the knee joint was maximally extended from a 90° flexed position in a sitting position, and the maximum value was adopted. Walking speed, one-leg standing, and 5CS tests were measured to determine physical function. Comfortable walking speed was measured twice in a straight-line distance of 5 m at a preferred walking speed, and the average value was used. One-leg standing time was measured by standing on one leg on either side, and the maximum holding time was measured with a stopwatch. The maximum standing time on one leg was 60 s. The 5CS test was measured using a stopwatch when the participants stood from a 40 cm high chair five times with arms crossed in front of their chest. Frailty was assessed using the Japanese version of the Cardiovascular Health Study criteria [23], and robustness, pre-frailty, and frailty were determined based on grip strength, walking speed, physical activity level, and nutritional status. 

BMI was calculated as weight (kg) divided by height squared (m^2^), and leg circumference was measured at the point of maximum leg expansion using a tape measure. Skeletal muscle mass, skeletal muscle index, and phase angle were measured using a multi-frequency body composition analyzer (MC-780A-N, Tanita, Tokyo, Japan).

### 2.6. Determination of Sarcopenia and Respiratory Muscle Weakness and Each Group

**Figure 1:**
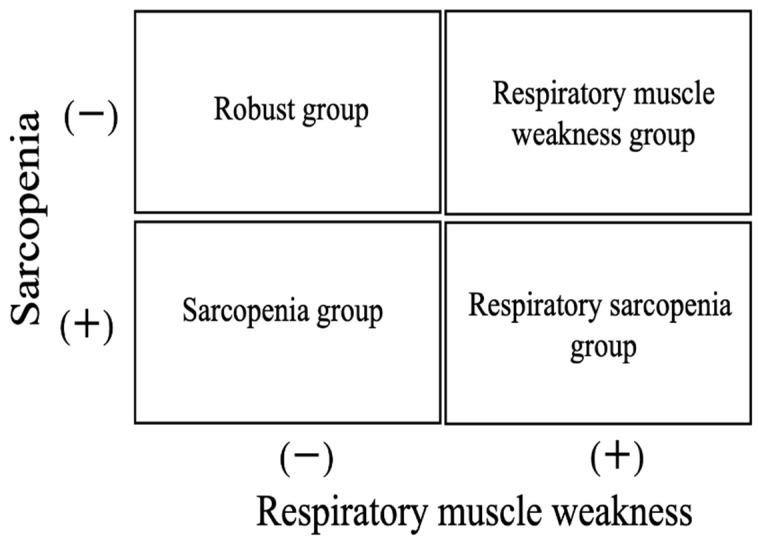
Determination of Sarcopenia and respiratory muscle weakness and each group.

Sarcopenia was judged based on the diagnostic criteria of the AWGS 2019 and classified as robust, sarcopenia, and severe sarcopenia [11], and in this study, sarcopenia was defined as “sarcopenia” and “severe sarcopenia”, as determined by AWGS 2019. In addition, respiratory muscle weakness was judged to be lower than that per the predicted respiratory muscle strength by Nishimura et al. [22]. The participants were classified into the “robust group” if they had neither sarcopenia nor respiratory muscle weakness, “sarcopenia group” if they had sarcopenia, “respiratory muscle weakness group” if they had respiratory muscle weakness, and “respiratory sarcopenia group” if they had both sarcopenia and respiratory muscle weakness (Figure 1).

### 2.7. Statistical Analysis

Data are presented as mean ± standard deviation. Multiple regression analysis with %MIP as the dependent variable and age, gender, and each group as independent variables was performed for the difference in mean deference of respiratory muscle strength among the three groups. The median and interquartile range were used when data did not show a normal distribution, a one-way analysis of variance was used to compare integer data among the four groups, and the χ^2^ test was used for categorical values. Differences were considered statistically significant at *p* < 0.05. All analyses were performed using IBM SPSS Statistics for Windows, version 21.0 (IBM Corp., Armonk, NY, USA).

## 3. Results

### 3.1. Basic Attributes of the Participants and Classification into Each Group

The basic attributes of the participants are shown in Table 1. Their mean age was 76.7 ± 5.9 years, and the proportion of female participants was 84.6%. The mean number of years of education was 13.1 ± 2.6 years, and the mean total KCL score was 5.5 ± 3.6 points. In total, 38 participants (32.5%) were classified into the robust group, 64 (54.7%) into the respiratory muscle weakness group, and 14 (12%) into the respiratory sarcopenia group. The sarcopenia group was excluded from statistical analyses because only one participant (0.8%) was in this category.

### 3.2. Mean Deference of Respiratory Muscle Strength among the Three Groups

**Figure 2:**
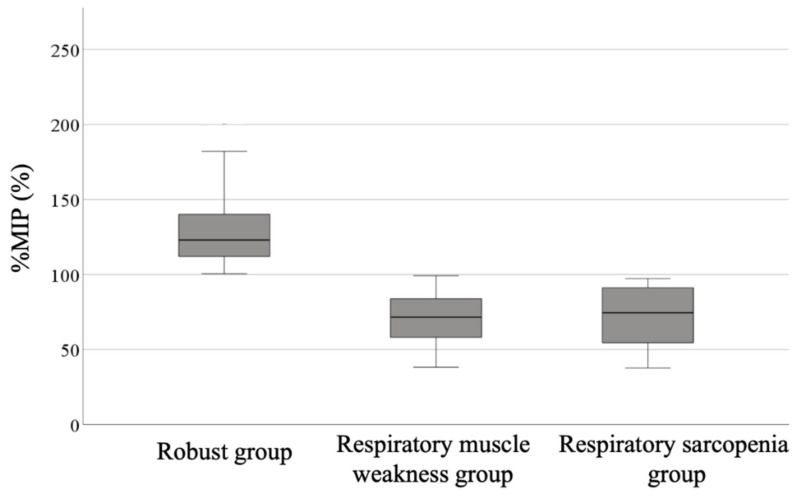
Mean deference of respiratory muscle strength among the three groups. %MIP, % maximum inspiratory pressure.

The %MIP was 128.9 ± 22.9% in the robust group, 70.9 ± 17.5% in the respiratory muscle weakness group, and 72.1 ± 21.3% in the respiratory sarcopenia group (Figure 2). Multiple regression analysis showed that when the robust group was used as a reference, the cointegration coefficient of the respiratory muscle weakness group was −58.15 (95% confidence interval (CI): −6618 to −50.12) and that of the respiratory sarcopenia group was −57.04 (95% CI: −71.29 to −42.79).

### 3.3. Basic Attributes, Physical Function, Body Composition, Oral Function, and Respiratory Muscle Strength among the Three Groups

The results of one-way analysis of variance for basic attributes, limb muscle strength, physical function, oral function, and cognitive function among the robust, respiratory muscle weakness, and respiratory sarcopenia groups are shown in Table 2. BMI was significantly lower in the respiratory muscle weakness and respiratory sarcopenia groups than in the robust group, and leg circumference was significantly lower in the respiratory sarcopenia group than in the robust group. Regarding body composition, skeletal muscle mass, skeletal muscle index, and phase angle were significantly lower in the respiratory sarcopenia group than in the robust group. The prevalence of frailty was significantly higher in the respiratory sarcopenia group. Physical function was significantly lower in the respiratory sarcopenia group than in the robust group in terms of grip strength, knee extensor strength, usual gait speed, chair stand, and one-leg standing time. Participants in the respiratory muscle weakness group had a significantly lower usual gait speed than those in the robust group. For oral function, those in the respiratory sarcopenia group had significantly lower values for “pa” and “ta” than those in the robust group.

### 3.4. Kihon Checklist among the Three Groups

**Figure 3:**
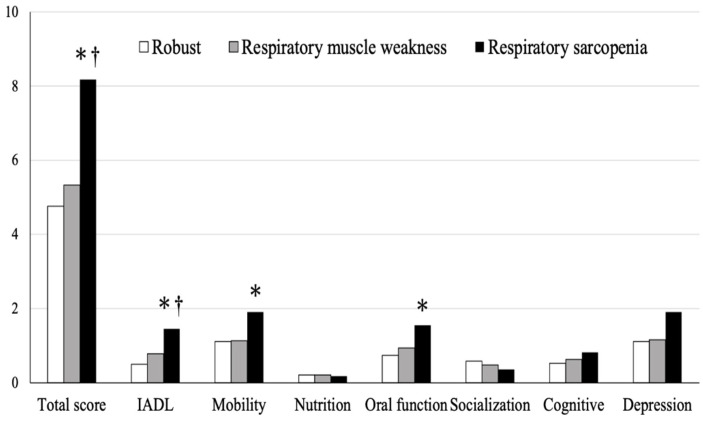
Comparison of the Kihon checklist among the three groups. * *p* < 0.05, vs. robust group, † *p* < 0.05, vs. respiratory muscle weakness group. IADL, instrumental activities of daily living.

A comparison of the KCL between the three groups is shown in Figure 3. The total score was significantly worse in the respiratory sarcopenia group than in the robust and respiratory muscle weakness groups, and the IADL items were significantly worse in the respiratory sarcopenia group than in the robust and respiratory muscle weakness groups. In addition, the respiratory sarcopenia group had significantly worse mobility and oral function values than the robust group.

## 4. Discussion

This study is the first to investigate the interrelationship between sarcopenia and respiratory muscle weakness in community-dwelling older adults. The main findings of this study were that (1) almost all community-dwelling older adults with sarcopenia had decreased respiratory muscle strength, and (2) physical function, muscle strength, and skeletal muscle mass of the group without sarcopenia but with decreased respiratory muscle strength were intermediate between those of the robust group and respiratory sarcopenia group. These results suggest that the development of systemic sarcopenia may be influenced in no small part by respiratory muscle weakness.

The prevalence of sarcopenia in the population of this study was 12.8%, which was similar to that reported in a systematic review and meta-analysis of the prevalence of sarcopenia in community-dwelling elderly (approximately 10%) [1,2]. Respiratory muscle weakness was observed in all but one of the participants with prevalent sarcopenia. In addition, there were a certain number of people with reduced respiratory muscle strength even without sarcopenia, and this result supports the possibility that sarcopenia and respiratory muscle weakness are related in no small part.

The aging process leads to a reduction in respiratory muscle strength. The causes vary but are believed to include muscle weakness and diaphragm atrophy [24], decreased cross-sectional area of the intercostal muscles, decreased curvature of the diaphragm due to structural changes in the chest wall, and decreased amplitude of the diaphragmatic action potential [25].

In addition, the mechanism of respiratory metabolic reflexes is known to be preceded by loss of muscle mass and weakness in the respiratory muscles such as the diaphragm and intercostal muscles, resulting in decreased exercise tolerance due to decreased oxygen-carrying capacity, skeletal muscle fatigue, and muscle weakness in limb skeletal muscles [16]. In fact, this respiratory muscle weakness may have caused metabolic reflexes that led to the development of sarcopenia in the participants of this study. Therefore, longitudinal evaluation is necessary to determine whether these groups with respiratory muscle weakness develop sarcopenia, and this is a subject for future study.

The participants in the respiratory muscle weakness group had a significantly lower BMI and a significantly lower gait speed, compared with the robust group. SMI, walking speed, and grip strength, which are diagnostic criteria for sarcopenia, all showed intermediate values between the robust group and the respiratory muscle weakness group, suggesting that respiratory muscle weakness slightly influences the development of sarcopenia.

Sarcopenia and respiratory muscle weakness are closely related to physical activity. According to the Kihon checklist of life functions in the elderly, the respiratory sarcopenia group had significantly lower IADL, motor function, and oral function than the robust group. Considering that the decline in motor function and IADL is directly related to the decline in physical activity, we cannot deny the possibility that physical activity decreased due to the decline in motor function and IADL. Although there was no statistically significant difference in the respiratory muscle weakness group, considering that it was between the robust and respiratory sarcopenia groups, it was suggested that a decline in IADL and motor function may also have had a small effect on respiratory muscle weakness and the development of sarcopenia. 

The main limitation of this study is that the sample size was not sufficient. In addition, 89% of the participants were females; thus, there was a gender bias. Furthermore, this was a cross-sectional study using measurements taken at a single point in time, and longitudinal studies are required to determine whether respiratory muscle weakness develops into sarcopenia, which is a future challenge.

## 5. Conclusions

Respiratory muscle weakness in the community-dwelling elderly group was observed in almost all cases of sarcopenia and was also observed in certain individuals without sarcopenia, suggesting that respiratory muscle weakness may precede sarcopenia. The physical function and skeletal muscle mass in the respiratory muscle weakness group were lower than those in the robust group and higher than those in the respiratory sarcopenia group, suggesting that physical activity decreased due to respiratory muscle weakness, which may have caused loss of skeletal muscle mass loss and physical function decline.

## Figures and Tables

**Table 1:** Basic information about the participants.

	Mean ± SD	Range
Age, year	76.7 ± 5.9	65–93
Female, %(n)	84.6 (99)	
Height, cm	154.9 ± 6.9	(134.0–175.0)
Weight, kg	52.6 ± 8.7	(34.0–72.8)
BMI, kg/m^2^	21.9 ± 3.2	(16.4–30.9)
Education history, year	13.1 ± 2.6	9–21
Medical history, %(n)		
Cancer	5.9 (7)	
Heart disease	7.7 (9)	
Stroke	3.4 (4)	
Diabetes	6.8 (8)	
Osteoporosis	12.8 (15)	
Osteoarthritis	15.4 (18)	

SD: standard deviation, BMI: body mass index.

**Table 2:** Basic attributes, physical function, body composition, oral function, and respiratory muscle strength of the participants compared by group.

	Robust Group (*n* = 38)	Respiratory Muscle Weakness Group (*n* = 67)	Respiratory Sarcopenia Group (*n* = 11)	*p* Value
Age, year	76.2 ± 5.6	76.7 ± 6.3	79.9 ± 4.7	0.185
sex, male/female, n	3/35	9/58	5/6 ** ^†^	<0.01
BMI, kg/m^2^	23.1 ± 3.3	21.6 ± 3.1 *	20.2 ± 1.9 *	<0.01
SMI	6.83 ± 0.71	6.60 ± 0.66	5.71 ± 0.62 **	<0.001
SMM, kg	16.47 ± 2.65	15.99 ± 2.84	13.84 ± 2.78 *	0.024
Phase angle, °	4.67 ± 0.44	4.48 ± 0.53	3.98 ± 0.66 **	<0.001
lower leg circumference, cm	35.4 ± 2.4	34.0 ± 3.0	32.0 ± 2.9 **	<0.01
Medical history, %(n)				
Cancer	5.3(2)	2.9(2)	27.3(3)	<0.01
Heart disease	10.5(4)	4.5(3)	9.1(1)	0.479
Stroke	0(0)	4.5(3)	9.1(1)	0.270
Diabetes	7.9(3)	7.5(5)	0(0)	0.635
Osteoarthritis	21.1(8)	11.9(8)	18.2(2)	0.449
J-CHS criteria, %(n)				
Robust	63.2(24)	50.7(34)	0.0(0)	<0.001
Pre-frail	34.2(13)	46.3(31)	63.6(7)	
Frail	2.6(1)	3.0(2)	36.4(4)	
MIP, cmH_2_O	−66.2 ± 15.1	−37.3 ± 12.9 **	−41.1 ± 14.2 **	<0.001
%MIP, %	128.9 ± 22.9	70.9 ± 17.5 **	72.2 ± 21.2 **	<0.001
Grip strength, kg	23.7 ± 5.4	22.5 ± 5.6	18.9 ± 6.2 *	0.043
Knee extensor strength, kg	26.3 ± 7.7	23.8 ± 8.0	19.3 ± 7.9 *	0.033
Usual gait speed, m/s	1.50 ± 0.23	1.35 ± 0.30 *	1.04 ± 0.26 **	<0.001
One leg standing time, s	39.2 ± 21.5	38.3 ± 22.6	22.6 ± 20.9	0.076
Chair stand, s	6.74 ± 1.67	7.30 ± 3.35	9.43 ± 2.79 *	0.025
Oral function				
Pa	6.73 ± 0.50	6.48 ± 0.67	6.20 ± 0.67 *	0.028
Ta	6.67 ± 0.59	6.45 ± 0.68	6.13 ± 0.87 *	0.049
Ka	6.27 ± 0.63	6.09 ± 0.70	5.96 ± 0.74	0.287
Moca-J	25.4 ± 3.3	23.7 ± 3.9	23.1 ± 5.6	0.064

Mean ± standard deviation * *p* < 0.05, vs. robust group, ** *p* < 0.01 vs. robust group, ^†^ *p* < 0.01 vs. Respiratory muscle weakness group. BMI, body mass index; SMI, skeletal muscle index; SMM, skeletal muscle mass; J-CHS, Japanese version of the Cardiovascular Health Study; MIP, maximum inspiratory pressure; Moca-J, Montreal cognitive assessment-J.

## Data Availability

The data that support the findings of this study are available from the corresponding author upon reasonable request.

## References

[1] Papadopoulou S.K., Tsintavis P., Potsaki P., Papandreou D. (2020). Differences in the Prevalence of Sarcopenia in Community-Dwelling, Nursing Home and Hospitalized Individuals. A Systematic Review and Meta-Analysis. J. Nutr. Health Aging.

[2] Shafiee G., Keshtkar A., Soltani A., Ahadi Z., Larijani B., Heshmat R. (2017). Prevalence of sarcopenia in the world: A systematic review and meta- analysis of general population studies. J. Diabetes Metab. Disord..

[3] Kitamura A., Seino S., Abe T., Nofuji Y., Yokoyama Y., Amano H., Nishi M., Taniguchi Y., Narita M., Fujiwara Y. (2021). Sarcopenia: Prevalence, associated factors, and the risk of mortality and disability in Japanese older adults. J. Cachexia Sarcopenia Muscle.

[4] Spira D., Buchmann N., Nikolov J., Demuth I., Steinhagen-Thiessen E., Eckardt R., Norman K. (2015). Association of Low Lean Mass With Frailty and Physical Performance: A Comparison Between Two Operational Definitions of Sarcopenia-Data From the Berlin Aging Study II (BASE-II). J. Gerontol. A Biol. Sci. Med. Sci..

[5] Tsekoura M., Kastrinis A., Katsoulaki M., Billis E., Gliatis J. (2017). Sarcopenia and Its Impact on Quality of Life. Adv. Exp. Med. Biol..

[6] Beaudart C., Reginster J.Y., Petermans J., Gillain S., Quabron A., Locquet M., Slomian J., Buckinx F., Bruyère O. (2015). Quality of life and physical components linked to sarcopenia: The SarcoPhAge study. Exp. Gerontol..

[7] Woo J., Leung J., Morley J.E. (2015). Defining sarcopenia in terms of incident adverse outcomes. J. Am. Med. Dir. Assoc..

[8] Delmonico M.J., Harris T.B., Lee J.S., Visser M., Nevitt M., Kritchevsky S.B., Tylavsky F.A., Newman A.B., Health A., Body Composition S. (2007). Alternative definitions of sarcopenia, lower extremity performance, and functional impairment with aging in older men and women. J. Am. Geriatr. Soc..

[9] Beaudart C., Zaaria M., Pasleau F., Reginster J.Y., Bruyere O. (2017). Health Outcomes of Sarcopenia: A Systematic Review and Meta-Analysis. PLoS ONE.

[10] Cruz-Jentoft A.J., Bahat G., Bauer J., Boirie Y., Bruyere O., Cederholm T., Cooper C., Landi F., Rolland Y., Sayer A.A. (2019). Sarcopenia: Revised European consensus on definition and diagnosis. Age Ageing.

[11] Chen L.K., Woo J., Assantachai P., Auyeung T.W., Chou M.Y., Iijima K., Jang H.C., Kang L., Kim M., Kim S. (2020). Asian Working Group for Sarcopenia: 2019 Consensus Update on Sarcopenia Diagnosis and Treatment. J. Am. Med. Dir. Assoc..

[12] Enright P.L., Kronmal R.A., Manolio T.A., Schenker M.B., Hyatt R.E. (1994). Respiratory muscle strength in the elderly. Correlates and reference values. Cardiovascular Health Study Research Group. Am. J. Respir. Crit. Care Med..

[13] Nagano A., Wakabayashi H., Maeda K., Kokura Y., Miyazaki S., Mori T., Fujiwara D. (2021). Respiratory Sarcopenia and Sarcopenic Respiratory Disability: Concepts, Diagnosis, and Treatment. J. Nutr. Health Aging.

[14] Kera T., Kawai H., Hirano H., Kojima M., Watanabe Y., Motokawa K., Fujiwara Y., Ihara K., Kim H., Obuchi S. (2019). Definition of Respiratory Sarcopenia With Peak Expiratory Flow Rate. J. Am. Med. Dir. Assoc..

[15] Shin H.I., Kim D.K., Seo K.M., Kang S.H., Lee S.Y., Son S. (2017). Relation Between Respiratory Muscle Strength and Skeletal Muscle Mass and Hand Grip Strength in the Healthy Elderly. Ann. Rehabil. Med..

[16] Dempsey J.A., Romer L., Rodman J., Miller J., Smith C. (2006). Consequences of exercise-induced respiratory muscle work. Respir. Physiol. Neurobiol..

[17] Arai H., Satake S. (2015). English translation of the Kihon Checklist. Geriatr. Gerontol. Int..

[18] Fujiwara Y., Suzuki H., Yasunaga M., Sugiyama M., Ijuin M., Sakuma N., Inagaki H., Iwasa H., Ura C., Yatomi N. (2010). Brief screening tool for mild cognitive impairment in older Japanese: Validation of the Japanese version of the Montreal Cognitive Assessment. Geriatr. Gerontol. Int..

[19] Satake S., Shimokata H., Senda K., Kondo I., Toba K. (2017). Validity of Total Kihon Checklist Score for Predicting the Incidence of 3-Year Dependency and Mortality in a Community-Dwelling Older Population. J. Am. Med. Dir. Assoc..

[20] Yamada M., Arai H. (2015). Predictive Value of Frailty Scores for Healthy Life Expectancy in Community-Dwelling Older Japanese Adults. J. Am. Med. Dir. Assoc..

[21] Kojima G., Taniguchi Y., Kitamura A., Shinkai S. (2018). Are the Kihon Checklist and the Kaigo-Yobo Checklist Compatible With the Frailty Index?. J. Am. Med. Dir. Assoc..

[22] Nishimura Y., Maeda H., Tanaka K., Hashimoto A., Hashimotoo Y., Yokoyama M., Fukuzaki H. (1991). The Effect of Aging on Respiratory Muscle Function. Nihon Kyobusikkann Gakkai Zassi.

[23] Satake S., Arai H. (2020). The revised Japanese version of the Cardiovascular Health Study criteria (revised J-CHS criteria). Geriatr. Gerontol. Int..

[24] Elliott J.E., Greising S.M., Mantilla C.B., Sieck G.C. (2016). Functional impact of sarcopenia in respiratory muscles. Respir. Physiol. Neurobiol..

[25] Lalley P.M. (2013). The aging respiratory system--pulmonary structure, function and neural control. Respir. Physiol. Neurobiol..

